# Understanding the Effect of Diluents on Powder Flow and Tablet Properties of Poorly Compressible Black Pepper Extract Using Mixture Design Approach

**DOI:** 10.1155/ijfo/8655112

**Published:** 2025-12-05

**Authors:** Chaowalit Monton, Jirapornchai Suksaeree

**Affiliations:** ^1^ Drug and Herbal Product Research and Development Center, College of Pharmacy, Rangsit University, Pathum Thani, Thailand, rsu.ac.th; ^2^ Department of Pharmacognosy, College of Pharmacy, Rangsit University, Pathum Thani, Thailand, rsu.ac.th; ^3^ Medicinal Cannabis Research Institute, College of Pharmacy, Rangsit University, Pathum Thani, Thailand, rsu.ac.th; ^4^ Department of Pharmaceutical Chemistry, College of Pharmacy, Rangsit University, Pathum Thani, Thailand, rsu.ac.th

**Keywords:** design of experiment, dibasic calcium phosphate, microcrystalline cellulose, optimization, spray-dried lactose

## Abstract

The application of black pepper extract in dietary supplement formulations is often limited by its inherently poor compressibility, which renders direct compression into tablets problematic. Therefore, the selection of appropriate diluents to enhance the compressibility of black pepper extract is essential. This study employed an augmented simplex lattice design comprising three diluents: microcrystalline cellulose (MCC) PH102, spray‐dried lactose (SDL), and dibasic calcium phosphate (DCP). The powder flowability of the black pepper aqueous extract, both alone and in combination with the specified diluents, was evaluated using various parameters: angle of repose, bulk density, tapped density, compressibility index, and Hausner ratio. Subsequently, 600 mg tablet formulations containing 100 mg of black pepper aqueous extract were prepared under a compression pressure of 1500 psi, and the physical characteristics of the resulting tablets, including hardness, friability, and disintegration time (DT), were measured. The findings indicated that SDL exhibited superior flowability compared with MCC and DCP. For tablet formulations, MCC emerged as the most effective diluent, demonstrating optimal parameters: high hardness, low friability, and minimal DT. Specifically, the optimal tablet formulation achieved a hardness ranging from 8.63 to 9.04 kgf, with friability between 0.07% and 0.29%, and DT between 2.73 and 4.42 min. Verification of these results demonstrated predictive accuracy with low residual values. In summary, this investigation highlights the pivotal role of diluents in enhancing the compressibility and overall tablet characteristics of black pepper aqueous extract. Among the evaluated diluents, MCC PH102 proved to be the most effective in promoting the desired tablet properties, thereby facilitating the direct compression of this otherwise poorly compressible substance.

## 1. Introduction

The utilization of black pepper extract in dietary supplements and therapeutic formulations has gained attention in recent years. Its application is sometimes limited because of its poor compressibility, presenting challenges for direct tablet compression [1]. The physical properties of black pepper extract, such as poor flowability and compressibility, can negatively impact both the tablet manufacturing process and the quality of the final tablets, which are important for ensuring optimal piperine release, bioavailability, and patient adherence. The granulation method has the potential to address the issues associated with the poor compressibility of certain substances [2–4], including black pepper extract. However, it still presents significant challenges, primarily due to the increased complexity of the process and the necessity for more sophisticated equipment compared with the direct compression method. Furthermore, the wet granulation method may be unsuitable for moisture‐sensitive or thermolabile ingredients [5, 6]. To address these challenges, the introduction of appropriate diluents for direct compression is essential.

Black pepper is renowned for its active constituent piperine, which has been widely studied for its bioenhancing effects on various nutraceuticals and pharmaceuticals [7, 8]. Piperine is known to enhance the bioavailability of several nutritionally significant compounds through multiple mechanisms, including modification of intestinal absorption and inhibition of drug‐metabolizing enzymes [8, 9]. Furthermore, the beneficial properties of black pepper extend beyond nutrient absorption; its potential capacity to enhance digestive health makes it a valuable addition to dietary formulations [10]. Therefore, evaluating the compressibility of black pepper extract combined with various diluents is paramount in maximizing its technological properties in tablet form.

The choice of diluents is critical in improving powder flow properties, which directly influence the efficiency of the manufacturing process [11, 12]. Microcrystalline cellulose (MCC) is widely recognized for its excellent binding and acceptable flow properties, making it a common choice in tablet formulations [13, 14]. In contrast, spray‐dried lactose (SDL) exhibits distinctive flow characteristics attributed to its spherical morphology, potentially enhancing the flowability of poorly compressible materials such as black pepper extract [15, 16]. Dibasic calcium phosphate (DCP) also serves as a diluent with established compressibility profiles, yet its effectiveness compared with MCC and SDL remains to be fully elucidated, specifically for black pepper applications [17, 18].

This study employs an augmented simplex lattice design to systematically evaluate the interactions between black pepper extract and the selected diluents. Parameters such as the angle of repose, bulk density, tapped density, compressibility index, and Hausner ratio will be assessed to determine powder flowability. Following this, tablet formulations will be produced under controlled conditions to measure physical properties such as hardness, friability, and disintegration time (DT), all critical for defining the quality and performance of the final dosage forms.

The results are expected to elucidate the role of diluents in enhancing the compressibility of black pepper extract, thereby improving tablet properties. Previous studies have highlighted the beneficial effects of MCC in producing tablets with high mechanical strength and low friability [1]. This investigation is expected to offer valuable insights into the formulation optimization of poorly compressible substances, potentially broadening the pharmaceutical application of black pepper extract. A comprehensive understanding of the interaction between diluents and black pepper extract is critical for the development of effective dietary supplements and therapeutic products, ultimately promoting improved accessibility and patient adherence.

## 2. Materials and Methods

### 2.1. Materials

Black pepper aqueous extract powder (1% piperine) was purchased from AP Operations Co. Ltd., Chonburi, Thailand. MCC (Comprecel M102) and SDL (SuperTab 11SD) were purchased from Maxway Co. Ltd., Bangkok, Thailand. DCP and magnesium stearate were purchased from Krungthepchemi Co. Ltd., Bangkok, Thailand. Talcum was purchased from Nitika Pharmaceutical Specialities Pvt. Ltd., Nagpur, India. Fumed silica was purchased from P.C. Drug Center, Bangkok, Thailand.

### 2.2. Scanning Electron Microscopy (SEM) Analysis of Diluents

The morphology of the diluents was analyzed using SEM (JSM‐7800F (Prime), JEOL Ltd., Tokyo, Japan). Before imaging, the samples were sputter‐coated with a thin layer of gold to enhance conductivity and image quality. SEM photomicrographs were obtained at a magnification of ×500 to observe surface characteristics and particle morphology in detail.

### 2.3. Determination of Particle Size and Particle Size Distribution of Diluents

Particle size and particle size distribution were determined using a laser diffraction technique with a Mastersizer 2000 instrument (Malvern Instruments, Worcestershire, UK) equipped with a Scirocco 2000 dry powder dispersion unit. The system utilized a red He‐Ne laser (*λ* = 633 nm) and a blue solid‐state light source for particle detection. The optical system had a beam length of 10.0 mm and was capable of analyzing particle sizes ranging from 0.02 to 2000 *μ*m. The dispersing medium was air, delivered at a pressure of 3.0 bar, with a feed rate set to 50%. The sample refractive index was specified as 1.480, and the laser power was maintained at 73.2 during measurement. Each sample was analyzed in triplicate.

### 2.4. Preparation of Black Pepper Extract Powder Mixture and Tablets

The combination of three diluents was formulated based on an augmented simplex lattice design using Design‐Expert software version 11 (Stat‐Ease, Inc., MN, United States). All procedures, from powder preparation and blending to tablet compression and evaluation, were conducted at an ambient temperature of 25°C–30°C and relative humidity below 70%. The mass ratios of MCC, SDL, and DCP for each formulation are presented in Tables 1 and 2. Each tablet contained 100 mg of black pepper extract as the active ingredient, 6 mg of magnesium stearate as a lubricant, 18 mg of talcum as an anti‐adherent, 6 mg of fumed silica as a glidant, and 470 mg of the designated diluent combination, resulting in a total tablet weight of 600 mg.

**Table 1:** Factors and responses for the augmented simplex lattice design varying the mass ratios of different diluents on flow properties.

**Std.**	**Run**	**Factors**			**Responses**				
		**Mass ratios**			**Flow properties (** **n** = 3**)**				
		**MCC**	**SDL**	**DCP**	**Angle of repose (°)**	**Bulk density (g/cm** ^**3**^ **)**	**Tapped density (g/cm** ^**3**^ **)**	**Compressibility index (%)**	**Hausner ratio**
11	1	0.33	0.33	0.33	39 ± 2.1	0.54 ± 0.01	0.75 ± 0.01	28 ± 0.8	1.38 ± 0.02
7	2	0.66	0.17	0.17	38 ± 1.1	0.49 ± 0.01	0.66 ± 0.01	25 ± 1.4	1.33 ± 0.03
2	3	0.00	1.00	0.00	34 ± 1.5	0.62 ± 0.01	0.72 ± 0.01	14 ± 2.0	1.16 ± 0.03
1	4	1.00	0.00	0.00	37 ± 1.2	0.46 ± 0.02	0.59 ± 0.00	22 ± 3.1	1.29 ± 0.05
4	5	0.50	0.50	0.00	33 ± 0.9	0.57 ± 0.02	0.68 ± 0.01	16 ± 2.5	1.20 ± 0.04
5	6	0.50	0.00	0.50	43 ± 1.9	0.55 ± 0.01	0.85 ± 0.02	36 ± 0.3	1.55 ± 0.01
12	7	0.33	0.33	0.33	43 ± 1.1	0.60 ± 0.03	0.85 ± 0.05	30 ± 2.0	1.43 ± 0.04
9	8	0.17	0.17	0.66	44 ± 1.6	0.59 ± 0.00	1.00 ± 0.00	41 ± 0.0	1.70 ± 0.00
10	9	0.33	0.33	0.33	42 ± 0.8	0.61 ± 0.02	0.87 ± 0.03	30 ± 2.1	1.42 ± 0.04
3	10	0.00	0.00	1.00	51 ± 1.1	0.54 ± 0.04	1.01 ± 0.00	46 ± 3.4	1.87 ± 0.12
8	11	0.17	0.66	0.17	34 ± 2.8	0.65 ± 0.04	0.84 ± 0.00	22 ± 5.0	1.29 ± 0.08
6	12	0.00	0.50	0.50	44 ± 0.8	0.63 ± 0.00	1.00 ± 0.00	38 ± 0.0	1.60 ± 0.00

*Note:* MCC, SDL, and DCP refer to microcrystalline cellulose PH102, spray‐dried lactose, and dibasic calcium phosphate, respectively. Runs 1, 7, and 9 are replicate runs at the center of the design.

**Table 2:** Factors and responses for the augmented simplex lattice design varying the mass ratios of different diluents on tablet properties.

**Std.**	**Run**	**Factors**			**Responses**		
		**Mass ratios**			**Tablet properties**		
		**MCC**	**SDL**	**DCP**	**Hardness (kgf,** **n** = 5**)**	**Friability (%, 6 tablets)**	**DT (min,** **n** = 3**)**
11	1	0.33	0.33	0.33	2.08 ± 0.11	3.75	1.59 ± 0.50
7	2	0.66	0.17	0.17	5.07 ± 0.47	0.31	0.61 ± 0.04
2	3	0.00	1.00	0.00	1.60 ± 0.36	12.8	26.7 ± 1.10
1	4	1.00	0.00	0.00	8.77 ± 0.30	0.06	4.63 ± 1.14
4	5	0.50	0.50	0.00	3.26 ± 0.33	0.75	0.64 ± 0.02
5	6	0.50	0.00	0.50	3.38 ± 0.41	1.21	0.97 ± 0.47
12	7	0.33	0.33	0.33	2.05 ± 0.55	3.66	5.90 ± 0.02
9	8	0.17	0.17	0.66	1.77 ± 0.33	8.71	1.09 ± 0.20
10	9	0.33	0.33	0.33	2.16 ± 0.23	2.90	2.25 ± 1.80
3	10	0.00	0.00	1.00	1.44 ± 0.25	12.80	125.17 ± 0.97
8	11	0.17	0.66	0.17	1.15 ± 0.14	21.98	3.78 ± 0.40
6	12	0.00	0.50	0.50	1.17 ± 0.34	18.23	16.57 ± 3.14

*Note:* MCC, SDL, and DCP refer to microcrystalline cellulose PH102, spray‐dried lactose, and dibasic calcium phosphate, respectively. Runs 1, 7, and 9 are replicate runs at the center of the design.

All components were passed through a 60‐mesh sieve, except for magnesium stearate, which was sieved through an 80‐mesh sieve. All ingredients, excluding magnesium stearate, were blended using the geometric dilution method, with each mixing step lasting 5 min. Magnesium stearate was incorporated in the final step and mixed for an additional 5 min. The prepared powder blends were evaluated for their flow properties before tablet compression.

For tablet compaction, 600 mg of each powder blend was compressed using a hydraulic press equipped with a pressure gauge, applying a force of 1500 psi. The die used in this study had an internal diameter of 12.7 mm. The resulting tablets were assessed for properties.

### 2.5. Evaluation of Powder Flow

The powder flow properties of mixtures containing black pepper extract, diluent(s), and other excipients were evaluated based on the angle of repose, bulk density, tapped density, compressibility index, and Hausner ratio. The angle of repose (*α*) was determined using the fixed funnel method. Briefly, 10 g of the powder mixture was gently poured through a glass funnel positioned at a height of 5 cm. The diameter and height of the resulting powder cone were measured (*n* = 3), and the angle of repose was calculated accordingly. Bulk density was determined by transferring the powder mixture into a graduated cylinder, followed by recording its mass (m) and initial volume (V_o_) (*n* = 3). The cylinder was then manually tapped until a constant volume was obtained, and the final volume (V_f_) was recorded. Tapped density was calculated based on these measurements (*n* = 3). The compressibility index and Hausner ratio were derived from V_o_ and V_f_. The equations used for the calculation of all powder flow parameters are presented in Equations (1)–(5).

(1) tanα=height/0.5 base

(2) Bulk density=m/Vo

(3) Tapped density=m/Vf

(4) Compressibility index=100×Vo−Vf/Vo

(5) Hausner ratio=Vo/Vf

### 2.6. Evaluation of Tablet Properties

The tablets were assessed for hardness, friability, and DT. Because of the limited availability of black pepper extract in this study, the number of tablets used for each test was adjusted. All evaluations were performed in accordance with a previous study [19]. Briefly, tablet hardness was measured for five tablets using a digital hardness tester (HD‐200N, Wenzhou Sundoo Instruments Co. Ltd., Zhejiang, China). Friability was assessed for six tablets using a friability tester (CS‐2, Tianjin Guoming Medicinal Equipment Co. Ltd., Tianjin, China) at 25 rpm for 4 min. DT was evaluated for three tablets using a disintegration tester (BJ‐2, Tianjin Guoming Medicinal Equipment Co. Ltd., Tianjin, China) with water as the medium at 37°C.

### 2.7. Modeling of Powder Flow and Tablet Properties

The three‐dimensional (3D) surface plots illustrating the effect of diluent combinations on powder flow characteristics and tablet properties were generated using Design‐Expert software. In this study, a comprehensive analysis of variance (ANOVA) was conducted in order to identify the significant parameters affecting the outlined properties. Following this, the design spaces corresponding to various formulations were meticulously constructed. Subsequently, to validate the predictive accuracy of the model, the optimal formulation identified within these design spaces was prepared for further empirical verification.

## 3. Results

### 3.1. Powder Flow Properties

Morphology and particle size of the particles directly influence powder flow properties. The morphology of the diluents was examined using SEM, while particle size and particle size distribution were determined using laser diffraction. SEM photomicrographs of each diluent, along with their corresponding particle size distribution curves, are presented in Figure 1. The morphology of MCC appeared fibrous, SDL exhibited a spherical shape, and DCP displayed a flake‐like and irregular morphology. Visual observation suggested that SDL had the largest particle size, followed by MCC and DCP, respectively. However, particle size distribution curves indicated that SDL exhibited a broader particle size distribution compared with the other diluents. Exact values of particle size and particle size distribution are provided in Table 3. Interestingly, the mean particle diameter (by volume) was highest for MCC, followed by SDL and DCP, which contrasts with the SEM observations that suggested SDL appeared largest. Span values, indicating the width of the particle size distribution, were highest for SDL, followed by DCP and MCC, which aligns with the particle size distribution curves in Figure 1.

Figure 1SEM photomicrographs of (a) MCC (microcrystalline cellulose PH102), (b) SDL (spray‐dried lactose), and (c) DCP (dibasic calcium phosphate), along with their corresponding particle size distribution plots.

**Figure:**
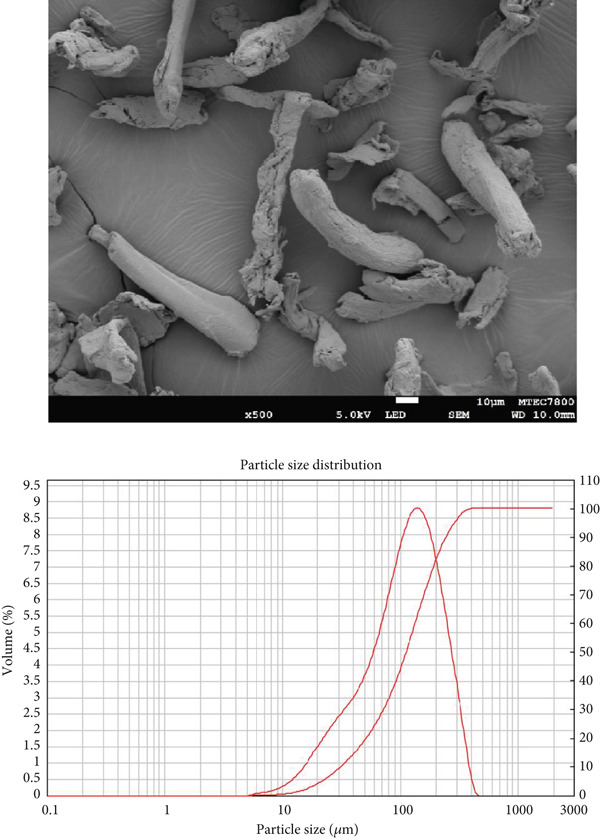
(a)

**Figure:**
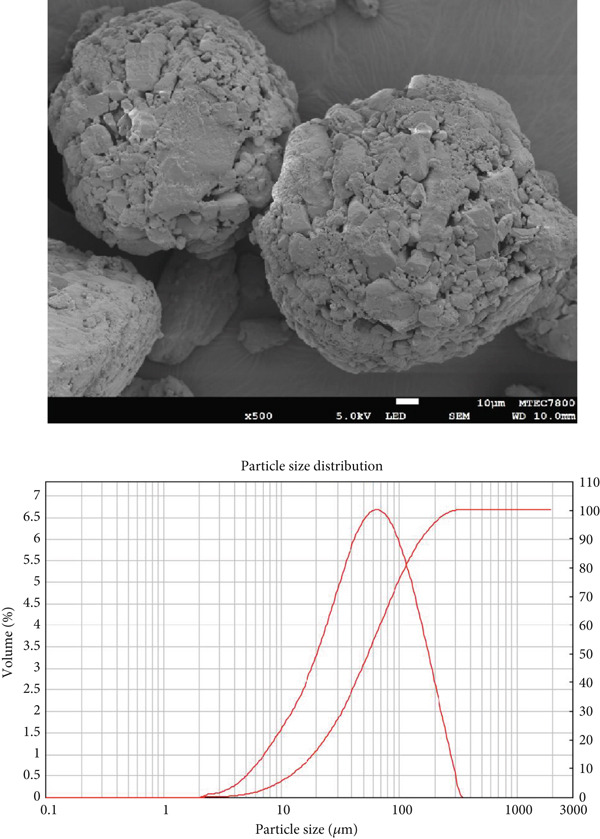
(b)

**Figure:**
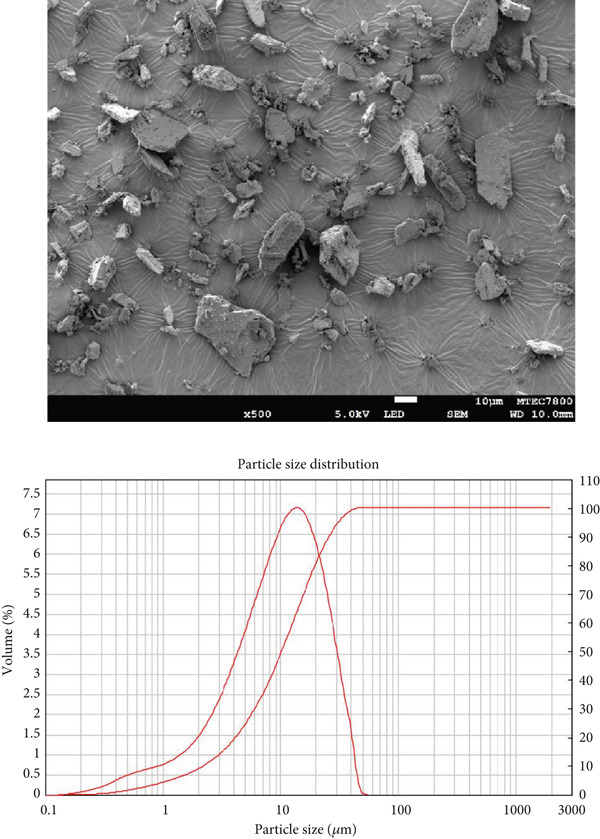
(c)

**Table 3:** Particle size and particle size distribution of diluents.

**Diluents** ^**a**^	**Diameter (D), *μ*m (** **n** = 3**)** ^**b**^				**Span** ^**c**^
	**Mean diameter (by volume)**	**D10**	**D50**	**D90**	
MCC	127.22 ± 0.47	31.30 ± 0.19	112.53 ± 0.58	244.95 ± 0.52	1.90
SDL	72.80 ± 0.48	14.40 ± 0.12	55.52 ± 0.19	157.72 ± 1.29	2.58
DCP	12.43 ± 0.04	2.21 ± 0.01	10.24 ± 0.04	26.04 ± 0.08	2.32

^a^MCC is microcrystalline cellulose PH102, SDL is spray‐dried lactose, DCP is dibasic calcium phosphate.

^b^D10, D50, and D90 are 10, 50, or 90 volumes % less than or equal to D, respectively.

^c^Span = (D90 − D10) / D50.

The powder flow properties of black pepper extract and the individual diluents used in this study were evaluated to characterize their inherent behaviors, with the results shown in Table 4. Assessing the flowability of each diluent before blending provided baseline information on their intrinsic properties. When the black pepper extract was later combined with these diluents and other pharmaceutical excipients, the flow characteristics of the final blends could be altered, and this baseline data helps to better interpret the behavior of the blended powders. Based on the angle of repose, black pepper extract exhibited poor flowability, whereas the compressibility index and Hausner ratio indicated very poor flow, in accordance with the United States Pharmacopeia (USP) classification. Among the diluents, MCC, SDL, and DCP demonstrated fair, good, and poor flow, respectively, based on angle of repose. In terms of compressibility index, MCC, SDL, and DCP exhibited very poor, passable, and very, very poor flow, respectively. According to the Hausner ratio, all three diluents were categorized as having very, very poor flow.

**Table 4:** Flow properties of black pepper extract and individual diluents.

**Sample** ^**a**^	**Angle of repose (°)**	**Bulk density** **(g/cm** ^**3**^ **)**	**Tapped density** **(g/cm** ^**3**^ **)**	**Compressibility index (%)**	**Hausner ratio**
Black pepper extract	48 ± 0.8	0.38 ± 0.01	0.58 ± 0.02	35 ± 3.6	1.54 ± 0.09
MCC	45 ± 1.5	0.33 ± 0.01	0.53 ± 0.01	37 ± 1.0	1.60 ± 0.03
SDL	35 ± 2.2	0.73 ± 0.23	0.96 ± 0.25	25 ± 4.0	1.68 ± 0.66
DCP	54 ± 3.6	0.56 ± 0.02	1.09 ± 0.03	49 ± 2.8	1.95 ± 0.11

*Note:* Data are presented as mean ± SD from triplicate experiments.

^a^MCC is microcrystalline cellulose PH102, SDL is spray‐dried lactose, DCP is dibasic calcium phosphate.

Powder flow of mixtures containing black pepper extract was modeled using an augmented simplex lattice design. The responses for each powder flow property are shown in Table 1, while 3D surface plots illustrating these properties are presented in Figure 2. Lower values of angle of repose, compressibility index, and Hausner ratio indicate better flowability. Accordingly, the surface plots revealed that mixtures containing SDL exhibited the best flow properties, followed by those containing MCC and DCP, respectively.

Figure 2Three‐dimensional (3D) surface plots of powder flow parameters: (a) angle of repose, (b) bulk density, (c) tapped density, (d) compressibility index, and (e) Hausner ratio. A, B, and C represent MCC (microcrystalline cellulose PH102), SDL (spray‐dried lactose), and DCP (dibasic calcium phosphate), respectively.

**Figure:**
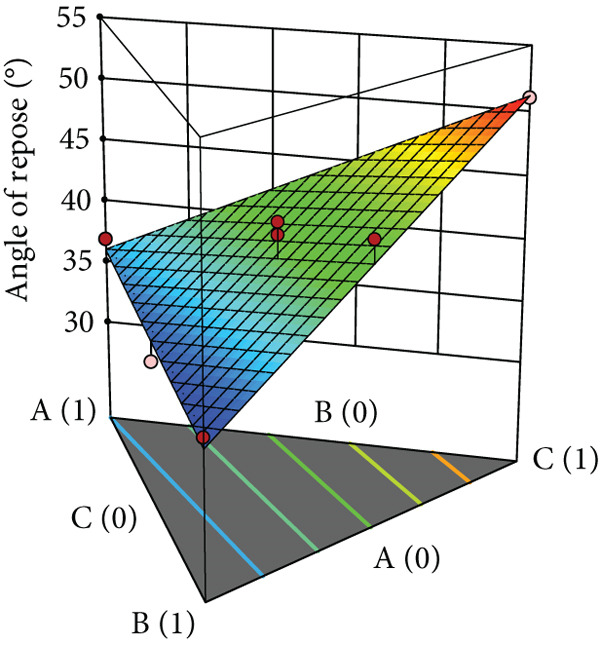
(a)

**Figure:**
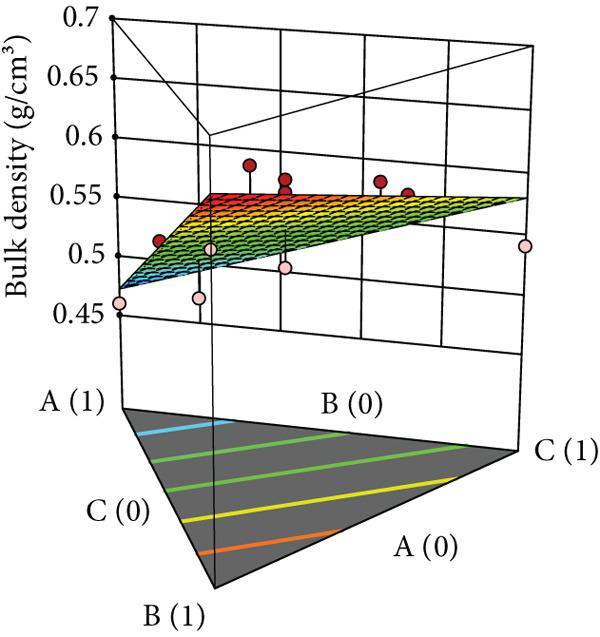
(b)

**Figure:**
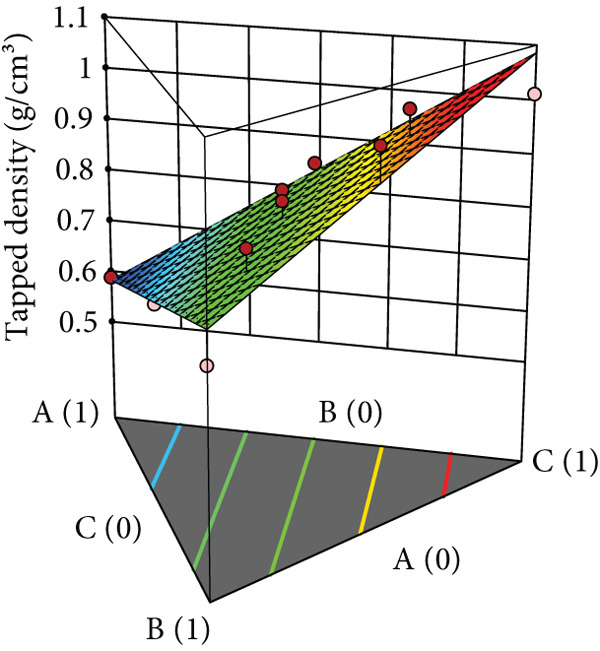
(c)

**Figure:**
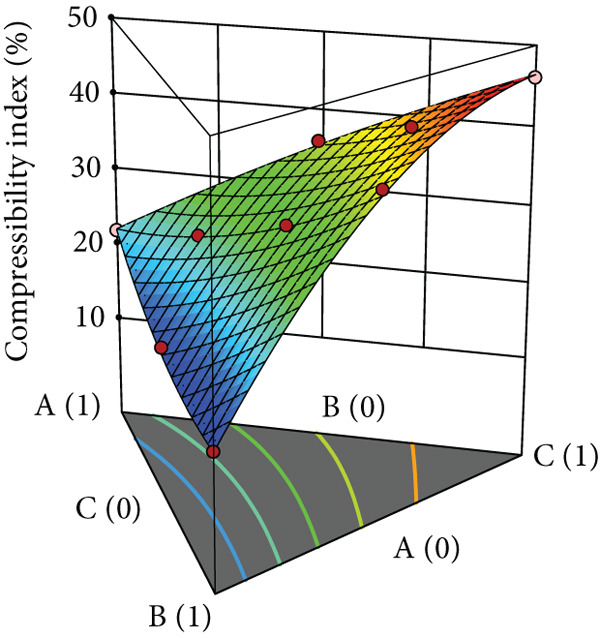
(d)

**Figure:**
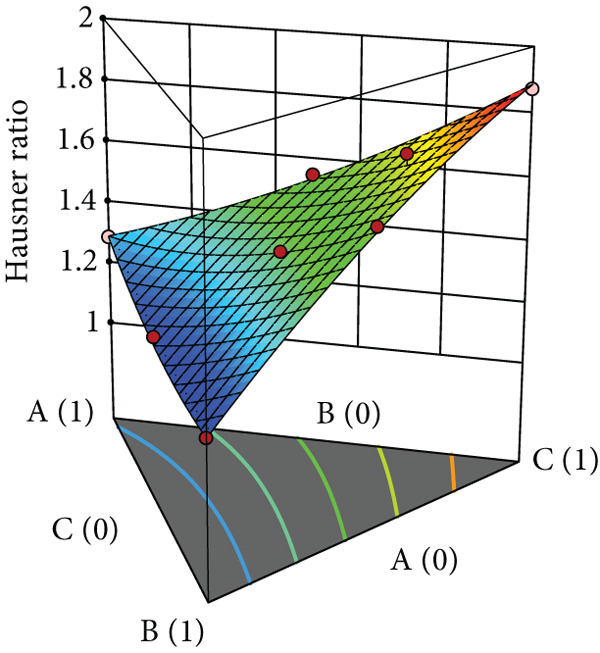
(e)

ANOVA results confirmed that the model was statistically significant, while the lack‐of‐fit test was not significant, indicating an adequate model fit. Angle of repose, bulk density, and tapped density conformed to a linear model, whereas compressibility index and Hausner ratio were best fitted by a quadratic model. All linear mixture terms were statistically significant. Furthermore, significant interactions were observed between MCC and SDL, and SDL and DCP for compressibility index (Table 5).

**Table 5:** ANOVA results and coefficients for flow properties.

**Source**	**Sum of squares**	**df**	**Mean square**	**F-value**	**p** **-value**	**Coefficient**
*Angle of repose*						
Model	279.0000	2	139.5000	40.9400	< 0.0001*	
Linear mixture	279.0000	2	139.5000	40.9400	< 0.0001*	36.17 (for A) 33.17 (for B) 51.17 (for C)
Residual	30.6700	9	3.4100			
Lack of fit	22.0000	7	3.1400	0.7253	0.6873	
Pure error	8.6700	2	4.3300			
Cor total	309.6700	11				
*Bulk density*						
Model	0.0263	2	0.0132	12.1100	0.0028*	
Linear mixture	0.0263	2	0.0132	12.1100	0.0028*	0.47 (for A) 0.66 (for B) 0.58 (for C)
Residual	0.0098	9	0.0011			
Lack of fit	0.0069	7	0.0010	0.6891	0.7030	
Pure error	0.0029	2	0.0014			
Cor total	0.0361	11				
*Tapped density*						
Model	0.1903	2	0.0952	30.3500	< 0.0001*	
Linear mixture	0.1903	2	0.0952	30.3500	< 0.0001*	0.59 (for A) 0.78 (for B) 1.09 (for C)
Residual	0.0282	9	0.0031			
Lack of fit	0.0200	7	0.0029	0.6897	0.7027	
Pure error	0.0083	2	0.0041			
Cor total	0.2186	11				
*Compressibility index*						
Model	1067.6600	5	213.5300	202.1900	< 0.0001*	
Linear mixture	1016.3300	2	508.1700	481.1700	< 0.0001*	22.22 (for A) 13.94 (for B) 46.40 (for C)
AB	7.7900	1	7.7900	7.3800	0.0348*	−11.98
AC	1.3200	1	1.3200	1.2500	0.3065	4.93
BC	43.7300	1	43.7300	41.4000	0.0007*	28.38
Residual	6.3400	6	1.0600			
Lack of fit	3.6700	4	0.9175	0.6881	0.6646	
Pure error	2.6700	2	1.3300			
Cor total	1074.0000	11				
*Hausner ratio*						
Model	0.4821	5	0.0964	115.6300	< 0.0001*	
Linear mixture	0.4744	2	0.2372	284.4900	<0.0001*	1.29 (for A) 1.16 (for B) 1.89 (for C)
AB	0.0024	1	0.0024	2.9100	0.1388	−0.21
AC	0.0022	1	0.0022	2.6200	0.1567	−0.20
BC	0.0035	1	0.0035	4.1400	0.0882	0.25
Residual	0.0050	6	0.0008			
Lack of fit	0.0036	4	0.0009	1.2900	0.4814	
Pure error	0.0014	2	0.0007			
Cor total	0.4871	11				

*Note:* A, B, and C are MCC (microcrystalline cellulose PH102), SDL (spray‐dried lactose), and DCP (dibasic calcium phosphate), respectively.

^*^Denotes a significant value.

The coefficients of each term reflect the extent of influence on powder flow properties. This analysis focuses specifically on angle of repose, compressibility index, and Hausner ratio, excluding bulk and tapped densities as they serve solely for calculating the latter two parameters. As lower values are desirable for all three responses, negative coefficient values indicate improved flowability, while positive values indicate reduced flowability. As shown in Table 5, SDL exhibited the lowest coefficient values among the linear terms for all parameters, indicating its minimal negative impact on flowability, followed by MCC and DCP, respectively. In contrast, DCP had the highest coefficient values, suggesting it exerted the greatest negative effect on flowability in the mixtures. For the compressibility index, the interaction between MCC and SDL improved flowability, followed by the interaction between MCC and DCP, while the interaction between SDL and DCP had the most detrimental effect. In terms of the Hausner ratio, interactions between MCC and SDL, and MCC and DCP reduced the ratio values, indicating enhanced flow, whereas the interaction between SDL and DCP negatively impacted powder flow.

### 3.2. Black Pepper Extract Tablet Properties

After evaluating the flowability of the powder mixture containing black pepper extract, it was compressed into tablets, and their properties—including hardness, friability, and DT—were assessed. The responses for each tablet property are shown in Table 2, while the surface plots are presented in Figure 3. Hardness increased with the use of MCC, whereas SDL and DCP yielded comparably low hardness, which further decreased with increasing levels of these excipients. For MCC, increasing its concentration reduced friability (Figure 3a). In contrast, increasing the level of SDL initially decreased friability, followed by an increase and subsequent decrease. MCC produced tablets with the lowest friability (Figure 3b). DCP exhibited an opposite trend to SDL. Moreover, MCC and SDL resulted in shorter DTs, while DCP produced tablets with the longest DT (Figure 3c).

Figure 3Three‐dimensional (3D) surface plots of tablet properties: (a) hardness, (b) friability, and (c) DT. A, B, and C represent MCC (microcrystalline cellulose PH102), SDL (spray‐dried lactose), and DCP (dibasic calcium phosphate), respectively.

**Figure:**
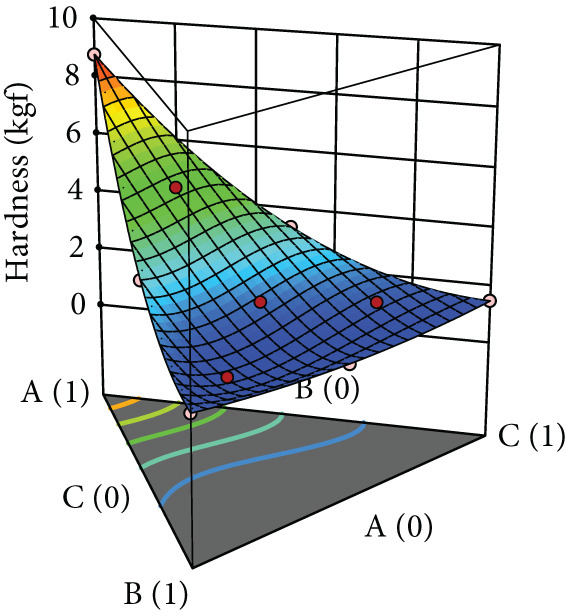
(a)

**Figure:**
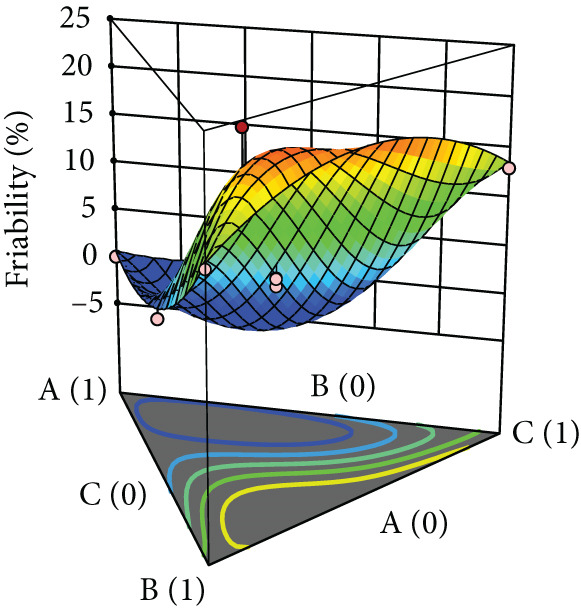
(b)

**Figure:**
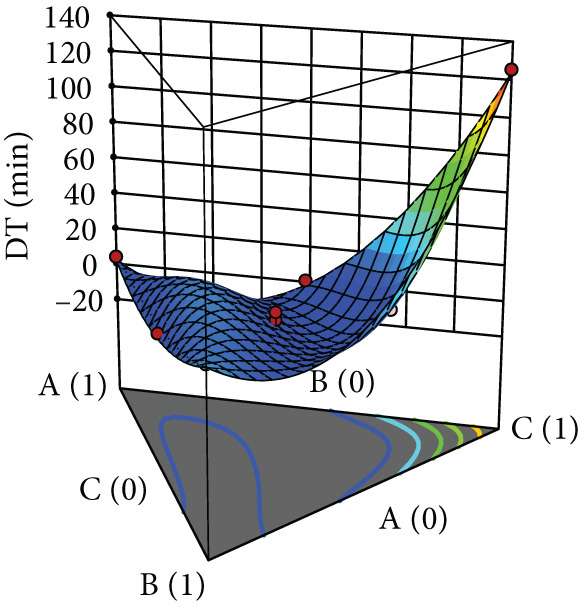
(c)

According to ANOVA, the models for hardness and DT were statistically significant, whereas the model for friability was not. The lack‐of‐fit test indicated that the model for hardness was not significant, suggesting an adequate model fit, while significant lack‐of‐fit was observed for friability and DT. Linear mixture terms were significant for all tablet properties. The significance of interaction and quadratic terms varied depending on the property. Specifically, interactions between MCC and SDL, and MCC and DCP were significant for hardness, whereas interactions between MCC and DCP, and SDL and DCP were significant for DT. Certain quadratic terms had a significant effect on hardness only (Table 6). According to the coefficient values, the terms that increased hardness included each linear component, MCC^2^ × SDL × DCP (A^2^BC), and MCC × SDL × DCP^2^ (ABC^2^). For friability, the terms that contributed to its reduction were the interactions between MCC and SDL, MCC and DCP, as well as the higher‐order terms A^2^BC and ABC^2^. In the case of DT, all interaction terms and the ABC^2^ term were associated with a reduction in DT (Table 6).

**Table 6:** ANOVA results and coefficients for the properties of black pepper extract tablets.

**Source**	**Sum of squares**	**df**	**Mean square**	**F-value**	**p** **-value**	**Coefficient**
*Hardness*						
Model	52.5300	8	6.5700	807.4200	< 0.0001*	
Linear mixture	45.6200	2	22.8100	2804.9500	< 0.0001*	8.78 (for A) 1.61 (for B) 1.45 (for C)
AB	2.4500	1	2.4500	301.6700	0.0004*	−7.66
AC	1.9700	1	1.9700	241.9200	0.0006*	−6.86
BC	0.0770	1	0.0770	9.4700	0.0543	−1.36
A^2^BC	0.1057	1	0.1057	12.9900	0.0366*	32.84
AB^2^C	0.2737	1	0.2737	33.6500	0.0102*	−52.84
ABC^2^	0.0237	1	0.0237	2.9200	0.1862	15.56
Residual	0.0244	3	0.0081			
Lack of fit	0.0179	1	0.0179	5.5500	0.1427	
Pure error	0.0065	2	0.0032			
Cor total	52.55	11				
*Friability*						
Model	570.6400	8	71.3300	4.0200	0.1401	
Linear mixture	384.4300	2	192.2100	10.8200	0.0425*	0.65 (for A) 13.39 (for B) 13.39 (for C)
AB	17.3300	1	17.3300	0.9755	0.3961	−20.35
AC	14.3300	1	14.3300	0.8071	0.4352	−18.51
BC	24.3000	1	24.3000	1.3700	0.3266	24.09
A^2^BC	17.6700	1	17.6700	0.9948	0.3921	−424.59
AB^2^C	69.8800	1	69.8800	3.9300	0.1416	844.41
ABC^2^	35.9100	1	35.9100	2.0200	0.2502	−605.31
Residual	53.2800	3	17.7600			
Lack of fit	52.8400	1	52.8400	242.3600	0.0041*	
Pure error	0.4361	2	0.2180			
Cor total	623.9200	11				
*DT*						
Model	13481.4300	8	1685.1800	20.2700	0.0155*	
Linear mixture	6006.6700	2	3003.3400	36.1200	0.0080*	3.37 (for A) 25.44 (for B) 123.91 (for C)
AB	177.6200	1	177.6200	2.1400	0.2400	−65.15
AC	2845.9200	1	2845.9200	34.2300	0.0100*	−260.77
BC	2461.3100	1	2461.3100	29.6000	0.0122*	−242.51
A^2^BC	507.0300	1	507.0300	6.1000	0.0901	2274.58
AB^2^C	155.7900	1	155.7900	1.8700	0.2645	1260.82
ABC^2^	655.6400	1	655.6400	7.8900	0.0674	−2586.50
Residual	249.4200	3	83.1400			
Lack of fit	238.6400	1	238.6400	44.2800	0.0218*	
Pure error	10.7800	2	5.3900			
Cor total	13730.8500	11				

*Note:* A, B, and C are MCC (microcrystalline cellulose PH102), SDL (spray‐dried lactose), and DCP (dibasic calcium phosphate), respectively.

^*^Denotes a significant value.

**Figure 4:**
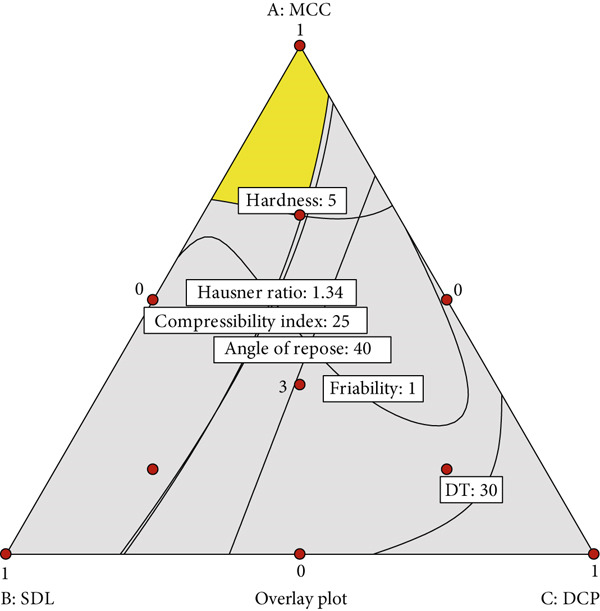
Design space representing the region where the angle of repose does not exceed 40°, the compressibility index is not more than 25, the Hausner ratio is not more than 1.34, tablet hardness is at least 5 kgf, friability does not exceed 1%, and DT is within 30 min. MCC, SDL, and DCP refer to microcrystalline cellulose PH102, spray‐dried lactose, and dibasic calcium phosphate, respectively.

The design space, defined as the region where the angle of repose does not exceed 40°, the compressibility index is not more than 25, the Hausner ratio is not more than 1.34, tablet hardness is at least 5 kgf, friability does not exceed 1%, and DT is within 30 min, is shown in Figure 4. Within this design space, formulations containing a higher proportion of MCC appeared to promote better overall properties.

MCC appeared to independently confer superior powder flowability and tablet characteristics. Therefore, a formulation of black pepper extract utilizing MCC alone as the diluent was prepared to evaluate the predictive accuracy of the model. Verification data are presented in Table 7. Tablet characteristics from three independent batches showed low residuals, indicating high predictive accuracy of the model.

**Table 7:** Verification results expressed as predicted values, experimental values, and their residuals.

**Parameters**	**Predicted values**	**Batch 1**		**Batch 2**		**Batch 3**	
		**Experimental values (** **n** = 3 **)**	**Residual** ^**a**^	**Experimental values (** **n** = 3**)**	**Residual** ^**a**^	**Experimental values (** **n** = 3**)**	**Residual** ^**a**^
Hardness (kgf)	8.78	9.04 ± 0.48	0.26	8.63 ± 0.42	−0.15	8.83 ± 0.29	0.05
Friability (%)	0.65	0.07	−0.58	0.11	−0.54	0.29	−0.36
DT (min)	3.37	2.98 ± 0.87	−0.39	4.42 ± 0.33	1.05	2.73 ± 0.98	−0.64

^a^Residual = experimental value – predicted value.

## 4. Discussion

This study utilized black pepper extract at a dosage of 100 mg per tablet, equivalent to 1 mg of piperine. This dosage is within the daily maximum allowable limit of 5 mg piperine for use in dietary supplement products in Thailand. However, the extract demonstrated poor compressibility, necessitating the application of high compression force to achieve adequate tablet compaction [1]. Moreover, the present study revealed that black pepper extract exhibited poor flowability as indicated by the angle of repose, and very poor flowability as determined by the compressibility index and Hausner ratio. Therefore, improvement of its flowability and compressibility was addressed by incorporating MCC, SDL, and DCP as excipients in the formulation of black pepper extract, owing to their distinct properties in enhancing flowability and compressibility, particularly in formulations involving poorly compressible materials.

This study demonstrated that individual diluents exhibited varying degrees of powder flowability. Notably, when these diluents were blended with other ingredients, including black pepper extract, the overall powder flow was improved. For example, in the case of MCC, the angle of repose decreased from 45^°^ ± 1.5^°^ to 37^°^ ± 1.2^°^, the compressibility index improved from 37*%* ± 1.0*%* to 22*%* ± 3.1*%*, and the Hausner ratio decreased from 1.60 ± 0.03 to 1.29 ± 0.05. Similar trends were observed with SDL and DCP. This improvement can be attributed to the presence of additional ingredients such as fumed silica and magnesium stearate, which are known to enhance powder flow properties [20–22].

The powder mixture containing SDL exhibited the best flowability compared with those containing MCC and DCP. This can be attributed to the spherical shape and larger particle size of SDL, which facilitates improved flow characteristics. MCC also demonstrated better flowability than DCP, likely due to its relatively larger particle size. In contrast, the smaller particle size of DCP may contribute to tighter packing and increased interparticulate friction, thereby hindering powder flow [23].

The observation that small fibrous‐shaped materials exhibit a larger effective volume compared with larger spherical materials can be attributed to several key factors related to particle morphology and agglomeration behavior. Firstly, fibrous particles possess a high aspect ratio, meaning their length is significantly greater than their diameter. This high aspect ratio results in a visually larger appearance; however, conventional size measurement techniques such as laser diffraction may lead to misinterpretation because these methods often average the dimensions of irregularly shaped particles. This can result in an inflated size measurement for fibrous particles compared with spherical ones, as the latter typically have a more uniform cross‐sectional area. The behavior of smaller fibrous particles can align in a manner that maximizes their effective volume when dispersed, leading to larger volumetric assessments despite reduced dimensions in any single direction. Additionally, agglomeration behavior is critical. Fibrous particles may tend to form clusters or networks because of their elongated shapes and surface interactions, which can increase the apparent size when measured using techniques sensitive to bulk volume. In contrast, larger spherical particles are less likely to aggregate to the same degree, resulting in more accurate size measurements that tend to be smaller. Moreover, the packing characteristics differ significantly between fibrous and spherical particles. Fibrous particles often create more complex arrangements that fill available spaces more efficiently compared with spherical particles, leading to a higher effective volume when analyzed collectively. This interaction with their surroundings can affect their perceived effective diameter during size analysis, further contributing to the larger size evaluation of fibrous materials [24–30].

Bulk and tapped densities of MCC (0.33 ± 0.01 and 0.53 ± 0.01 g/cm^3^) closely matched those reported previously (0.34 and 0.48 g/cm^3^). In contrast, SDL in the present study exhibited higher values (0.73 ± 0.23 and 0.96 ± 0.25 g/cm^3^) compared with the earlier report (0.60 and 0.71 g/cm^3^). DCP showed lower bulk and tapped densities (0.56 ± 0.02 and 1.09 ± 0.03 g/cm^3^) than those previously reported (0.92 and 1.17 g/cm^3^) [31]. This may be attributed to differences in their source, moisture content, particle size, and other material‐specific factors.

According to friability results, several formulations exhibited fragile characteristics, with some breaking completely. However, for modeling purposes, exact values must be used. Therefore, only the weight of compact particles—excluding loose powder—was measured after the friability test. This approach may have limited the study, as the method for determining the threshold size of compacts could have introduced variability that contributed to the model not being significant, with lack‐of‐fit exceeding pure error.

In the case of DT, the replicates showed low variability. Although the predicted model aligned well with the model points, indicating a significant overall fit, the variation among actual data points was considerably larger than the variation observed among the center points. This discrepancy led to a statistically significant lack‐of‐fit [32].

SDL generally produces tablets with lower hardness or tensile strength compared with plastically deforming excipients like MCC. This is mainly due to SDL’s brittle fracture behavior, which limits interparticle bonding during compression [33]. Its spherical particle shape reduces surface contact and mechanical interlocking, while limited plasticity hinders cohesive bond formation [34]. Although fragmentation creates new surfaces, these are often insufficient to compensate for the lack of plastic deformation [33].

During the DT determination, the authors observed that the formulation containing DCP as the sole diluent exhibited a prolonged DT, despite having low hardness and high friability. In contrast to literature reports indicating excellent flowability of DCP [31], the DCP used in this study demonstrated poor flow properties. This discrepancy may be due to differences in the grade or particle size of the DCP, with smaller particles potentially contributing to reduced flowability. Additionally, the inherently hydrophobic nature of DCP may have contributed to its poor disintegration in aqueous media [31].

Tablet properties indicated that MCC was the most suitable diluent for the poorly compressible black pepper extract, consistent with findings from the preparation of fingerroot extract tablets [35]. In a previous study, black pepper extract tablets were developed using MCC and DCP as diluents, where a high compression force (2000 psi) was applied to achieve desirable tablet properties [1]. DCP demonstrated low compactibility [36], resulting in high friability [37]. Based on the authors’ experience, several herbal formulations were optimized at a compression force of 1500 psi. Such high compression forces are typically employed when poor compressibility is observed, as reported in a previous study [19].

MCC is a versatile excipient widely used in tablet formulations, particularly in direct compression tablets, to enhance the ability of active pharmaceutical ingredients with poor compressibility [38–40]. Its exceptional compactibility and compressibility are attributed to its plastic deformation behavior, rod‐shaped particle morphology, high surface area, and low bulk density [19, 38, 41, 42]. However, MCC of varying grades sourced from different manufacturers exhibits significant differences in particulate and powder characteristics [43, 44]. During compression, MCC particles undergo plastic deformation, promoting the formation of tight junctions between adjacent cellulose molecules and enhancing hydrogen bond formation. This process also maximizes the bonding surface area, contributing to increased mechanical strength [45–47]. Additionally, MCC’s high compactibility arises from solid bridge formation and mechanical interlocking among its elongated particles. As the proportion of MCC in the formulation increases, the tablet’s mechanical strength correspondingly improves [14, 38]. Although rod‐shaped particles typically exhibit poor flowability, they demonstrate good compactibility. A lower bulk density generally corresponds to a higher specific surface area and a more porous internal structure, facilitating interaction with the active ingredient during compression and deformation [14, 38]. MCC also enhances liquid penetration into the tablet matrix, improving both diffusion and capillary action [14, 48], which contributes to its function as a disintegrant. The inclusion of MCC improves the tableting process, enabling the production of tablets with desirable qualities such as high tensile strength, acceptable DT, and low friability [14, 38, 49, 50].

This study demonstrated that MCC was the most effective diluent in improving the flowability and compressibility of black pepper extract, resulting in tablets with desirable mechanical and disintegration properties. The enhancement in tablet quality can be attributed to the plastic deformation and high bonding capacity of MCC, which facilitate the formation of strong interparticulate bonds during compression. Although the present study focused on physical tablet characteristics, it is important to note that the selection of diluents may also influence piperine release and bioavailability, which warrants further investigation. Additionally, long‐term storage stability and moisture sensitivity of the optimized formulations were not assessed in this study. These factors are important for ensuring consistent performance of MCC‐based tablets under practical manufacturing and storage conditions and will need to be investigated in subsequent studies. Overall, the key message is that MCC PH102 is the most effective diluent for improving both the flowability and compressibility of black pepper extract, enabling the production of tablets with desirable mechanical and disintegration properties suitable for dietary supplement applications.

## 5. Conclusions

This study demonstrated that the inherent poor compressibility of black pepper extract can be effectively addressed through the strategic selection of suitable diluents. Using an augmented simplex lattice design, the compressibility and flow properties of black pepper extract were significantly influenced by the type of diluent incorporated. While SDL exhibited superior flowability, MCC PH102 produced tablet formulations with optimal characteristics, including high hardness, low friability, and acceptable DT. The optimal formulation achieved hardness values between 8.63 and 9.04 kgf, friability between 0.07% and 0.29%, and DT ranging from 2.73–4.42 min. These findings were supported by predictive accuracy and low residual errors. Overall, MCC PH102 emerged as the most effective diluent, enabling the successful direct compression of black pepper extract and offering a promising approach for the formulation of plant‐derived dietary supplements.

## Conflicts of Interest

The authors declare no conflicts of interest.

## Author Contributions

**Chaowalit Monton:** conceptualization, methodology, formal analysis, investigation, project administration, resources, writing – original draft, and writing – review and editing. **Jirapornchai Suksaeree:** methodology, formal analysis, investigation, and writing – original draft.

## Funding

No funding was received for this manuscript.

## Data Availability

Data is available from the corresponding author upon request.
